# Vestibular Schwannoma Leading to Contacting Aneurysm Rupture: An Unusual Presentation

**DOI:** 10.7759/cureus.32716

**Published:** 2022-12-19

**Authors:** Renata Marques, João Nogueira, Maura Cambango, Frederica Coimbra, João Saraiva, Nubélio Duarte, Ângelo Carneiro, Miguel Afonso Filipe

**Affiliations:** 1 Neurosurgery Department, Hospital de Braga, Braga, PRT; 2 Neuroradiology Department, Hospital de Braga, Braga, PRT

**Keywords:** vestibular schwannoma, subarachnoid hemorrhage, contact aneurysm, cerebellopontine angle, aneurysm

## Abstract

Subarachnoid hemorrhage (SAH) is a rare manifestation of brain tumors, being even rarer in vestibular schwannomas. We report the second case of a posterior circulation aneurysm close to the tumor capsule, responsible for SAH as an initial manifestation of a vestibular schwannoma. A 67-year-old female was admitted to the Emergency Department with sudden onset of nausea and headache. A diagnosis of a SAH and a tumor of the right cerebellopontine angle was made. An angiography showed an aneurysm in the dependency of the right anterior inferior cerebellar artery (AICA), juxtaposed to the tumor capsule. The patient underwent surgery, the tumor was removed, and the aneurysm was treated. This case highlights that SAH in patients with vestibular schwannoma may originate from a contact aneurysm. Although exceedingly rare, surgeons should consider this scenario in vestibular schwannoma presenting with SAH, and angiography is important for its diagnosis.

## Introduction

Subarachnoid hemorrhage (SAH) as the initial presentation of a tumor is an uncommon event. The tumors that bleed most often are astrocytomas, oligodendrogliomas, metastatic tumors, meningiomas, choroid plexus papillomas, and hemangioblastomas [1,2].

The vestibular schwannoma is a benign tumor, which arises from Schwan cells usually in the vestibular division of the VIII cranial nerve. Its incidence rounds 11-13 cases per million every year and comprises about 6% to 10% of intracranial tumors [3,4]. The common initial symptoms of a vestibular schwannoma are hearing loss, tinnitus, ataxia, vertigo, headache, and facial numbness [4]. Vestibular schwannomas presenting with SAH on admission are extremely rare.

Cases of SAH in patients with vestibular schwannomas and concomitant aneurysms have been previously reported, the majority unruptured and in the anterior circulation [5,6]. To our knowledge, this is the second case of a posterior circulation aneurysm in the vicinity of a vestibular schwannoma causing a SAH, and the first to report it in a branch of the anterior inferior cerebellar artery (AICA).

## Case presentation

**Figure 1:**
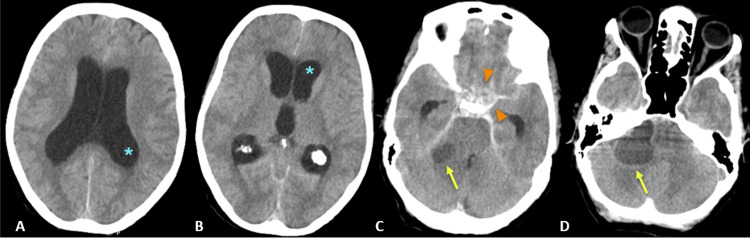
Axial cranial CT images on admission. (A, B) Images showing acute hydrocephalus (blue asterisks indicating dilated ventricles); (C) image revealing subarachnoid hemorrhage in basal cisterns (orange arrowheads) and a right cerebellopontine angle lesion (yellow arrow); (D) right cerebellopontine angle lesion (yellow arrow).

A 67-year-old woman, with past a medical history of stage IIIC breast cancer, stage 5 chronic kidney disease of undetermined etiology, and hypertension, was admitted to the Emergency Department with sudden onset of nausea and intense headache. On physical examination, she was agitated and disoriented, without focal neurological deficits. A cranial unenhanced computed tomography (CT) was performed revealing a spontaneous SAH in the basal and suprasellar cisterns, as well as sylviSylvianures and convexity sulci. Additionally, an expansive cystic lesion in the right cerebellopontine angle plus active supratentorial hydrocephalus were found (Figures 1A-1D).

**Figure 2:**
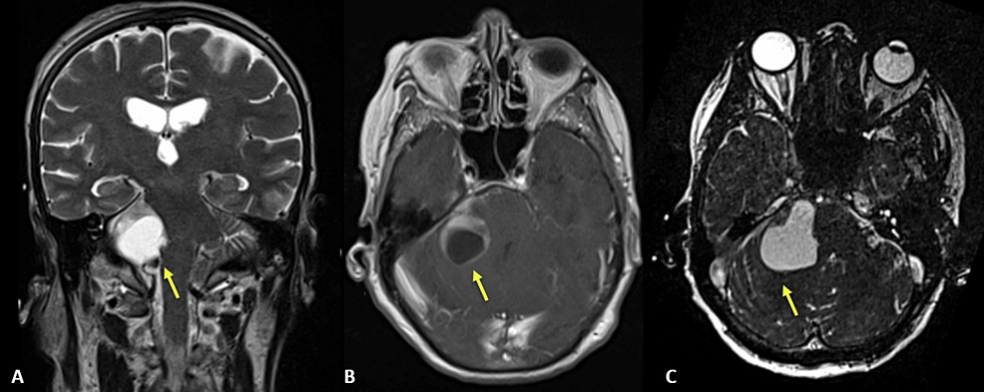
Cranial MRI showing apparently cystic cerebellopontine angle lesion, causing brainstem compression and IV ventricle collapse. (A) Coronal T2; (B) gadolinium-enhanced axial T1; (C) axial CISS sequence. Yellow arrow indicating the lesion.

In order to understand the bleeding source, a cranial CT angiography was performed, which did not reveal aneurysms or vascular malformations. While in the Emergency Department, the patient’s level of consciousness worsened, and an emergent external ventricular drain (EVD) was placed. The patient recovered and was admitted to the neurocritical care unit. On the following day, she underwent a cerebral angiography with catheterization of the right and left internal carotid arteries and the left vertebral artery, which ruled out aneurysms or vascular malformations. A cranial magnetic resonance imaging (MRI) was also performed to characterize the right cerebellopontine lesion, confirming an apparently cystic lesion with a small solid component measuring 3.7 cm in diameter, whose diagnostic hypotheses were metastases, meningioma or cystic hemangioblastoma (Figures 2A-2C).

**Figure 3:**
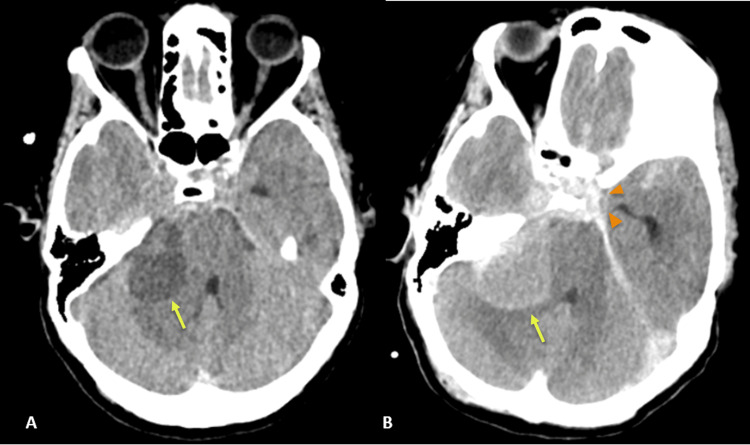
Cranial CT before and after EVD replacement. (A) Cranial CT before EVD replacement showing low density right cerebellopontine angle lesion (yellow arrow); (B) cranial CT after EVD replacement, revealing an increased density of the cerebellopontine lesion (yellow arrow) and additional blood in basal cisterns (orange arrowheads), reflecting re-bleeding.

On the sixth day of hospitalization, the patient externalized the EVD with the need for replacement. A postoperative CT scan confirmed correct EVD positioning but showed increased lesion density and additional blood in basal cisterns, reflecting possible re-bleeding (Figures 3A, 3B).

**Figure 4:**
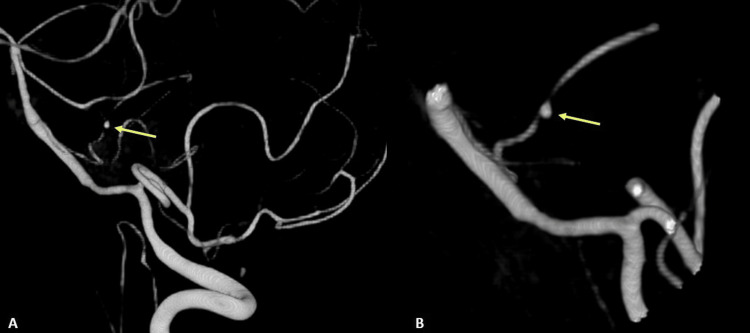
Angiography images revealing a small aneurysm in the vicinity of the tumor area. (A) AICA aneurysm (~1mm, yellow arrow); (B) close-up image of the aneurysm (yellow arrow).

Thus, a new angiography was performed, this time with the catheterization of the four vessels, revealing a small distal AICA pseudoaneurysm (Figures 4A, 4B).

**Figure 5:**
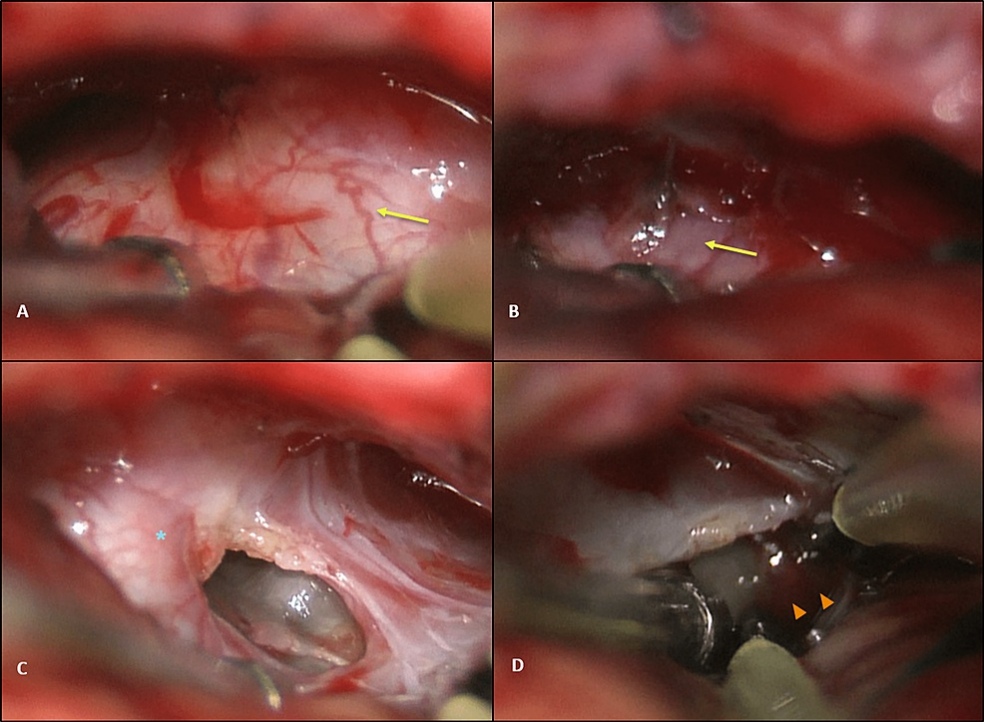
Intraoperative images of the tumor. (A, B) Solid tumor (yellow arrow); (C) thick capsule (blue asterisk); (D) blood clots inside the tumor (orange arrowheads).

It also showed mild to moderate vasospasm in posterior circulation. On the 12th day after admission, the patient underwent a right retrosigmoid craniotomy to excise the expansive lesion. Unexpectedly, the tumor was not cystic as believed based on preoperative imaging exams, but solid, with clots and liquefied hematic content reflecting previous bleeding. The lesion was extensively adherent to the VII cranial nerve (Figures 5A-5D).

**Figure 6:**
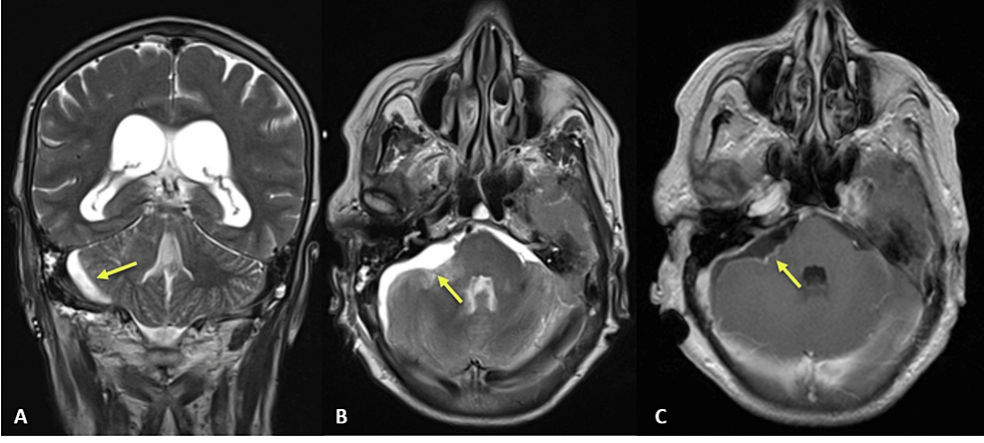
Post-operative cranial MRI, showing gross removal of the lesion. (A) Coronal T2; (B) axial T2; (C) gadolinium-enhanced axial T1. Yellow arrow indicating surgical site.

In the final phase of tumor removal, a small aneurysm was identified in the right AICA, in relation to that seen on angiography. It displayed bleeding during gentle manipulation before being excluded with bipolar coagulation. The remaining AICA was preserved, without noticeable infarct areas. Postoperative cranial MRI showed complete tumor removal (Figures 6A-6C).

The histological result was compatible with a schwannoma. On the 27th day of hospitalization, due to the maintenance of hydrocephalus, a ventriculo-peritoneal shunt was placed. The patient had a favorable clinic evolution, with surgical sequelae of right facial peripheral palsy, with no other neurological deficits.

## Discussion

Tumor hemorrhage comprises 1%-11% of the causes of intracranial hemorrhage and may manifest as SAH, intratumoral hemorrhage with SAH, or intraparenchymal hemorrhage around the tumor [7,8]. There are 14 reported cases of SAH in association with large vestibular schwannomas (Table 1) [8-19].

**Table 1:** Vestibular schwannoma presenting with subarachnoid hemorrhage as reported in the literature. AICA - anterior inferior cerebellar artery; PICA – posterior inferior cerebellar artery.

First Author, year	Sex	Age	Tumor Characteristics	Contacting aneurysm
McCoyd, 1974 [9]	Female	64	Hemorrhagic neurinoma, 3,5cm.	No
Fine, 1977 [12]	Female	21	Large acoustic neurinoma.	No
Casella, 1979 [13]	Male	54	Hemorrhage around tumor.	No
Gleeson, 1978 [20]	Female	54	4cm acoustic neurinoma. Dense scarring surrounding and old hemorrhage inside.	No
Shephad, 1981 [14]	Male	37	Large neurinoma.	No
Shephad, 1981 [14]	Male	66	Large neurinoma (3cm). Tumor covered with blood clot.	No
Castillo, 1982 [15]	Male	61	3-4cm; Liquefying hematoma inside a yellowish rim tumor.	No
Majchrzak, 1985 [16]	Male	32	Vascularized neurinoma with focal necrosis.	No
Sasaki, 1985 [17]	Female	33	Large neurinoma.	No
Mirinov, 1986 [18]	Female	61	Large neurinoma.	No
Kodama, 1987 [8]	Female	66	Large neurinoma. Yellowish brown capsule. Blood clot inside a solid tumor.	PICA aneurysm
Arienta, 1988 [19]	Female	47	Large neurinoma with cystic formation inside.	No
Chu, 2007 [10]	Female	45	Bilateral neurinomas. Yellowish brown capsule with xanthochromic fluid inside.	No
Gavra, 2010 [11]	Female	18	Solid neurinoma with a blood infiltrated capsule.	No
Marques, 2022 (current)	Female	67	Large and solid vestibular schwannoma with blood clots inside.	AICA aneurysm

There is also mention of a small number of cases of vestibular schwannomas where unruptured aneurysms of the anterior circulation were concomitantly found [5,6]. However, only one case was reported in which the cause of the SAH was an aneurysm in a branch of the posterior inferior cerebellar artery (PICA), adjacent to the tumor capsule [8]. In this case, the rupture of a small aneurysm in a branch of the AICA juxtaposed to the tumor was the cause of the hemorrhage. This is supported by the angiographic confirmation of the aneurysm in the proximity of the tumor and the existence of mild to moderate imagiological vasospasm in the vessels of the posterior circulation. Furthermore, the intraoperative observation of organized clots and intratumoral liquefied blood next to this small aneurysm, and its active bleeding in the final stage of the surgery support this aneurysm as the source of SAH. The non-saccular aneurysm was only 1 mm and it was not in a bifurcation of vessels, which leads us to think that the tumor growth itself may have caused its formation through the induction of vascular fragility and/or its rupture [8]. The repetition of the angiography after the rebleeding was important in its diagnosis since it had not been identified in the first exam. We believe that a phenomenon of permeabilization of the aneurysm may have occurred, since the exams were six days apart, allowing the identification of the small aneurysm.

## Conclusions

In patients with vestibular schwannoma who present with concomitant bleeding, tumor hemorrhage should not be assumed as the sole hypothesis. Especially if there is an associated SAH, the patient should be investigated further. In this report, the key to the diagnosis was the high rate of suspicion, which led us to repeat the angiography, leading to the successful treatment of the patient. Thus, in a patient with a vestibular schwannoma presenting with SAH, the presence of an aneurysm near the tumor should be considered a possible cause of bleeding and the angiography is crucial.
